# Habituation of changed acoustics properties after canal wall down mastoidectomy

**DOI:** 10.1007/s00405-022-07652-9

**Published:** 2022-09-17

**Authors:** M. R. Zwemstra, P. Brienesse, M. J. F. de Wolf, E. van Spronsen

**Affiliations:** Department of Otorhinolaryngology, Location Academic Medical Center, Amsterdam University Medical Centers, Meibergdreef 9, 1105 AZ Amsterdam, the Netherlands

**Keywords:** Ear canal acoustics, Canal wall down, Hearing quality outcome

## Abstract

**Purpose:**

Our objective is to evaluate the effect of habituation on the altered ear canal acoustics after canal wall down mastoidectomy.

**Methods:**

A total of 11 participants with single sided dry cavities and normal contralateral ear canals with normal hearing thresholds were included in the study. Filtered sound fragments were created that simulate the different acoustic properties based on the participants Real Ear to Coupler Difference (RECD) of the cavity ear and the contralateral normal ear canal. These filtered sound fragments are presented to the cavity ear canal and the contralateral normal ear canal using inserts earphones. Participants performed a subjective quality assessment of the sound fragments using paired comparison with a seven-point scale (− 3 till + 3).

**Results:**

Mean assessment of sound quality revealed the following results; naturalness of sounds of the cavity ear canal is 0.29 (± 1.41; ns) in favour of the filtered sound fragment of the normal ear canal. Mean assessment of sharpness of sounds in the cavity ear canal is 1.55 (± 1.21, *p* = 0.55) in favour of the filtered sound fragment of the normal ear canal. Overall preference in the cavity ear canal was in favour of the normal ear canal acoustics 0.72 (SD ± 1.54 *p* = 0.224).

**Conclusions:**

Patients with cavity ear canals seem to habituate to their altered ear canal acoustics. Transforming the ear canal acoustics of the cavity ear to normal ear canal acoustics seem to sharpen the incoming sounds. Overall assessment of quality of sound of the normal ear canal acoustics is better than the cavity acoustics.

## Introduction

Canal wall down (CWD) mastoidectomy is a common procedure for cholesteatoma surgery [1]. After CWD surgery a mastoid cavity is created. Not infrequently, the creation of a radical cavity is followed by symptoms of dizziness, discharge or deafness. Patients also depend on their otolaryngologist for regular microscopic cleaning of the cavity [2].

A contradiction is present when regarding post-operative hearing results between canal wall up and canal wall down mastoidectomy surgery [3]. Self-reported hearing functioning was found to be worse after canal wall down mastoidectomy in comparison with canal wall up mastoidectomy [3]. Yet, hearing thresholds are not significantly different between them [3]. A possible explanation for this conundrum could be the altered ear canal acoustics. We have previously demonstrated that the ear canal acoustics are changed in a radical cavity [4–7]. Ear canal acoustics is the modulation of soundwaves in the ear canal [8]. The normal ear canal acts as a filter, to reduce low frequencies and enhance mid to high frequencies [9]. In case of a cavity condition, the acoustic properties shift toward an amplification of the soundwaves of low to mid frequencies, and a reduction in soundwaves of high frequencies [10]. Normal hearing subjects with normal ear canals are capable of accurately discriminating differences between the acoustic properties of different ear canals after surgical modulation [10]. The possible effect of habituation to the altered acoustic properties of a radical cavity has never been studied. In tinnitus patients, a model for habituation is described. The attention system in the frontal cortex suppresses the response from the amygdala and other nodes of the limbic system when mediating emotion [11]. Although the impact of tinnitus on quality of life is presumably much larger than altered acoustics, the underlying mechanisms of habituation could match.

Our objective is to study whether patients with a single sided radical cavity experience habituation of their altered ear canal acoustics. Our second objective is to study how normal ear canal acoustic presentation on the cavity ear would be perceived.

## Participants and methods

### Subjects

We included 11 individuals with a dry single sided radical cavity. Of these 11 individuals, 6 (55%) were female. The average age of all participants was 43.6 years (median 39, range 25–64 years). Seven individuals (63%) have a right sided cavity. The subjects had a cavity ear for an average of 21 years (median 26, range 7–33 years). Of these cavity ears, 2 (18%) had a history of revision surgery of their cavity (Table 1). Bone conductive tone thresholds of the cavity ear were 20 dB HL or better at 0.25, 0.5, 1, 2 and 4 dB and air conductive tone thresholds were better than 70 dB HL at 0.25, 0.5, 1, 2 and 4 dB. Pure air conductive thresholds of the contralateral ear were 20 dB HL or better at 0.25, 0.5, 1, 2 and 4 dB. All participants agreed to participate in the study. The study protocol was in accordance of the Helsinki declaration and was approved by the ethical review board. None of the authors has a conflict of interest.

**Table 1:** Baseline characteristics

	Total, *n* = 11
Mean age (years)	43.6 (25–64)
Sex	
Male	5 (45%)
Female	6 (55%)
Side of cavity	12 (100%)
Left side	4 (36%)
Right side	7 (63%)
Years with cavity	21 (7–33)
Revision surgery radical cavity*	2 (18%)

*No posterior canal wall reconstruction, previous revision surgery indicated because of persistent ear discharge

### Methods

In short, we created filtered sound fragments for each participant that simulate the different acoustic properties of the cavity ear and the contralateral normal ear canal based on the participants individual Real Ear to Coupler Difference (RECD). These filtered sound fragments are presented to the cavity ear canal and the contralateral ear canal. Participants performed a subjective quality assessment of the filtered sound fragments using paired comparison.

**Fig. 1:**
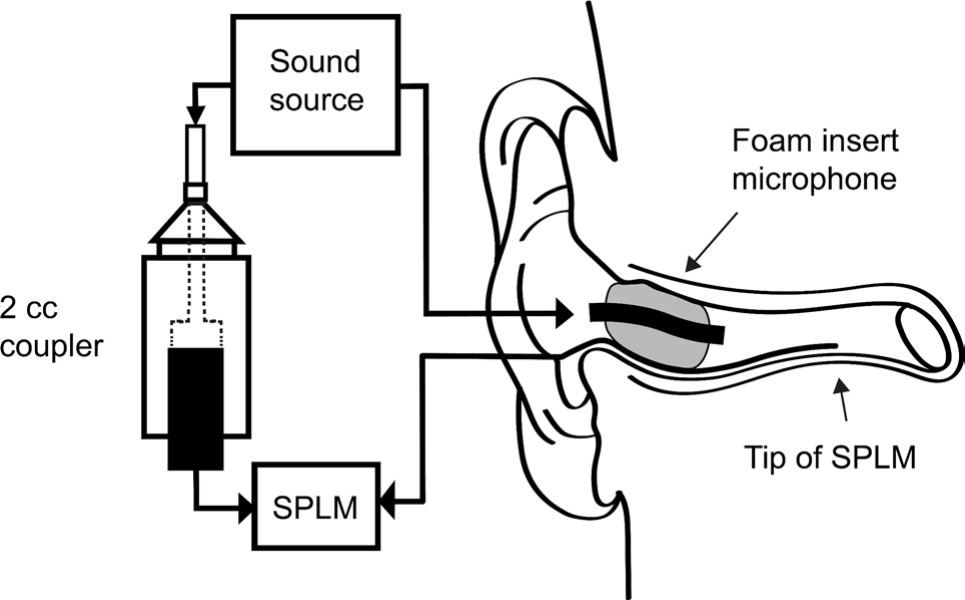
In this figure, a schematic representation of the real ear to coupler (RECD) measurement is displayed. The RECD accounts for the decibel difference across frequencies between sound pressure level (SPL) measured in the 2 cc coupler and the SPL measure in the participants individual ear, produced by the same transducer generating the same soundwave in decibels of a well-defined broadband sound stimulus. *SPLM* sound pressure level measure

To acquire the individual filtered sound fragments, we measured acoustic properties of the individuals ear canals via the RECD, being the frequency dependent gain in decibels (dB) of the soundwave from the insert microphone to the eardrum [12]. The RECD is measured using a microphone inserted in the ear canal, placed near the eardrum, measuring the frequency dependent gain of the soundwave in decibels of a well-defined broadband sound stimulus from the foam insert microphone in the ear canal (Fig. 1). All ear canals were dry and properly cleaned before measurement.

### Simulation of the acoustic properties of the individuals ear canals

Differences between individual RECDs represent differences in acoustic properties of individual ear canals. The acoustic effect of the measured acoustic properties of different ear canals can be simulated in the participants using a filter on the incoming sound stimulus. Therefore, we use the difference between the RECD of the individuals normal ear canal and the RECD of the cavity ear canal, using the REM module of the Affinity 2.0 Hearing Aid Analyzer platform (Interacoustics, Denmark). For our participants, this filtering results in the same distribution of sound pressure (acoustics) at the eardrum as in the contralateral ear canal, mimicking the acoustic effect of the cavity in a normal ear, and mimicking the acoustic effect of the normal ear in the cavity ear [6, 7, 10].

**Fig. 2:**
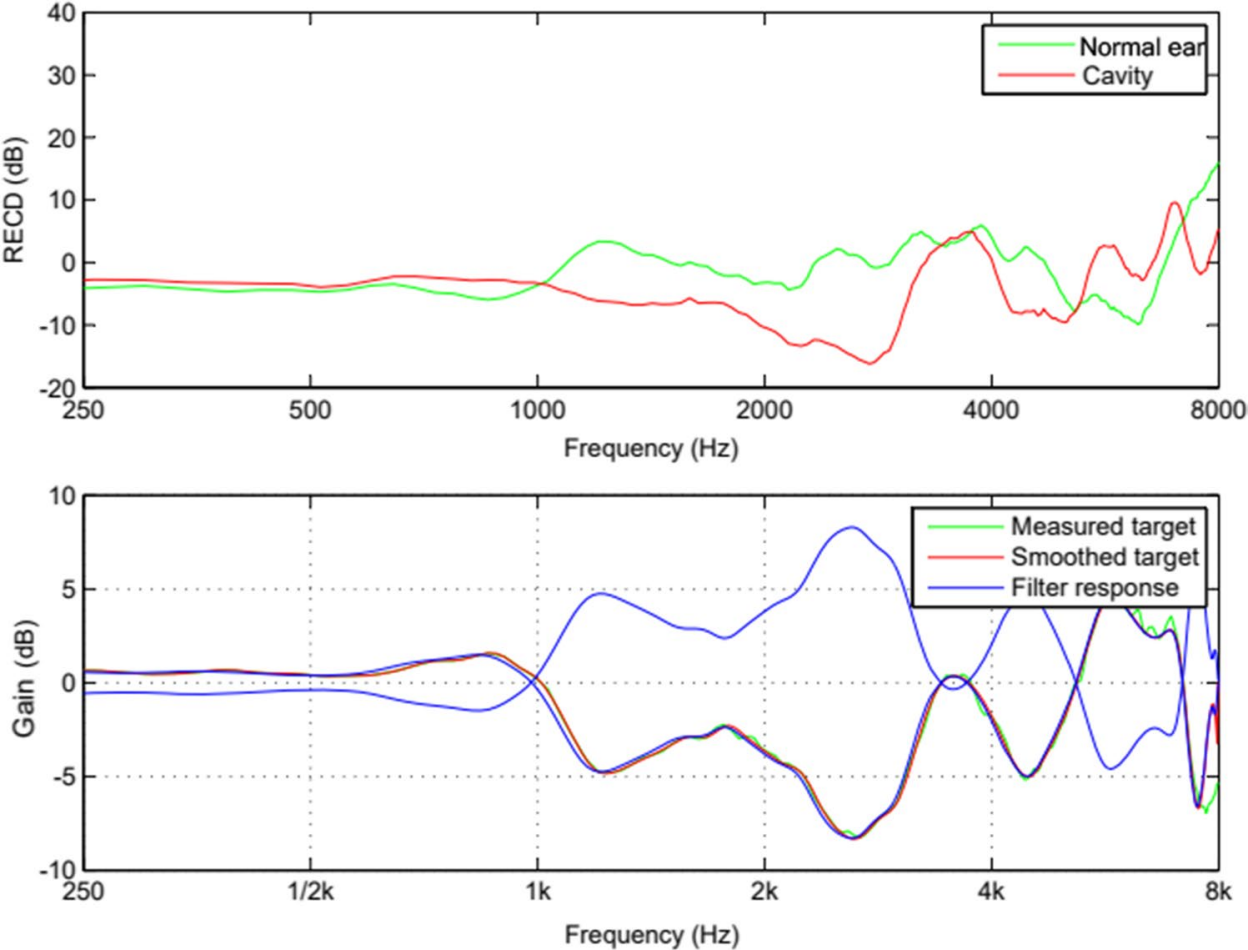
These graphs illustrate how we created our sound filters. In the top chart, the RECD of the participants normal ear canal is displayed in green and the participants cavity ear canal in red. In the bottom chart the green line displays the difference in RECD between the cavity canal and the normal ear canal. In red, the difference in RECD is smoothed. The blue line is the frequency dependent filter we used to mimic the cavity canal acoustics in the normal ear of this participant. The inverse of this filter is used to mimic the frequency dependent filter to mimic normal ear canal acoustics in the cavity ear canal. These filters are created for each participant. *RECD* real ear to coupler difference

We made recordings of Dutch speech (two male and two female speaker sentences based on the VU98 sentence material [13]), filtered to simulate the acoustic properties of the individuals contralateral ear canal. The acoustic filters, being the simulated conditions, were built on the differences between the individuals RECDs (Fig. 2).

### Perceptual evaluation

The perceptual evaluation experiment was performed with a paired comparison category rating between two fragments (‘A’ and ‘B’), according to ITU-T 1996 [14]. Participants were asked for each ear which fragment sounded more natural, more clear and better using a seven-point scale. Each filtered cavity sound fragment was compared to the sound fragment of the normal ear. These sound fragments are presented and compared, first in the normal ear and then in the cavity ear, using a foam insert microphone. All conditions were presented using four (Dutch) sentences by two male and two female voices and were measured twice for each: one time using the cavity filtered sound fragment as ‘A’ and the normal ear sound fragment as ‘B’, and one time in a reversed fashion. Thus, eight paired comparisons were presented in random order to assess naturalness of the sound for each ear canal. The same 8 paired comparisons were presented in random order to asses clearness of the sound, 8 paired comparisons to asses overall quality of the sound fragment, making a total of 48 paired comparisons for both ears. The paired comparison measurements are stated on a seven-point scale ranging from + 3 (the filtered cavity sentence sounds much more natural, clear or better than the normal ear) to − 3 (the normal ear sentence sounds much more natural, clear or better than the filtered cavity sentence). A score of 0 means there is no difference noticeable in naturalness, clearness or quality of sound.

All of the speech material was presented using in ear inserts at a level of 65 dB(A). In the cavity ear canal the microphone is amplified according to the NAL-NL1 rule to simulate the perception of 65 dB. Sound fragments were assigned to the subject in a random order alternately presented to the cavity ear canal and the normal ear canal.

### Statistical analysis

Data are expressed as numbers. Naturalness, clearness and quality of sound fragments presented to the normal ear and the cavity ear were compared using paired two tailed sample *t* test using IBM SPSS Statistics 26.

## Results

**Fig. 3:**
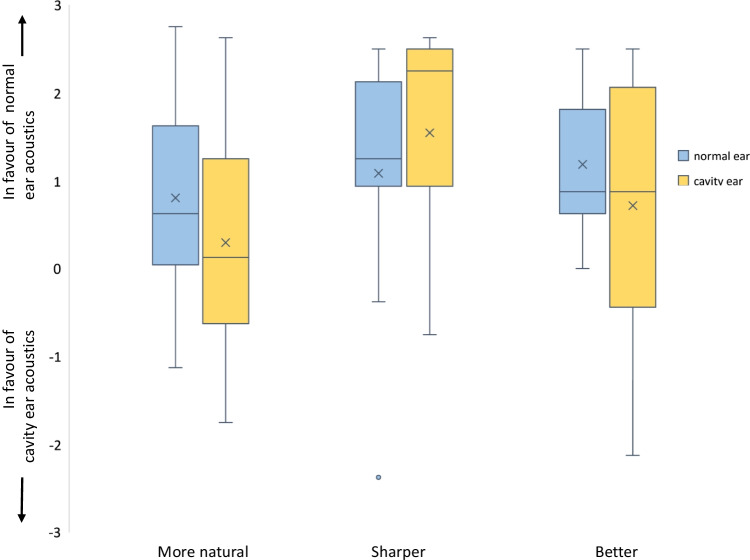
In this figure, the mean values for quality assessment of the sound fragments presented to the cavity ear canal and, respectively, the normal ear canal. They are presented as box-plots with 25th percentile and plotted on the horizontal axis for the assessment of naturalness, sharpness and overall quality (better) of sound. On the vertical axis the paired comparison outcome is displayed. Form 0 till + 3 represents an outcome in favour of the filtered sound fragments representing the normal ear canal acoustics. From 0 till − 3 represents an outcome in favour of the filtered sound fragment representing the cavity ear canal acoustics

Mean values for the measured sound qualities: naturalness-, clearness- and quality of sound fragments are presented in Fig. 3. Mean assessment of naturalness of the normal ear canal sound fragment is 0.8 (SD ± 1.12) in favour of the sound fragment of the normal ear canal. This indicates that, when the normal ear canal sound fragment is presented to the normal ear canal, the mean assessment of naturalness of the sound is slightly (+ 1) in favour of the normal ear canal acoustics. Mean assessment of naturalness the cavity ear canal sound fragment is marginally (0.29 (SD ± 1.41)) in favour of the filtered sound fragment of the normal ear canal. Differences in assessment of naturalness of the sound fragments between the normal ear canal and cavity ear canal were not significant (*p* = 0.321).

Mean assessment of sharpness of the sound fragments in the normal ear canal is 1.08 (SD ± 1.41) in favour of the normal ear canal sound fragment. Mean assessment of sharpness of the sound fragment in the cavity ear canal is 1.55 (SD ± 1.21). There was one outlier in the normal ear canal assessment with an assessment of -2.38, indicating for this individual the cavity sound fragment was much sharper than the normal ear sound fragment. Differences between mean assessment of sharpness of the sound fragments between the cavity- and normal ear canal were not significant (*p* = 0.055).

Mean assessment of overall quality of the sound fragment in the normal ear canal is 1.18 (SD ± 0.81), in favour of the normal ear canal sound fragment. Mean assessment of quality of the sound fragment in the cavity ear canal is 0.72 (SD ± 1.54), in favour of the normal ear canal sound fragment. Differences between the assessment of the quality of sound between the cavity- and normal ear canal were not significant (*p* = 0.224).

Mean assessment of naturalness, sharpness and quality of the sound fragment corresponded with the assessment of the male and female voices presenting the sound fragment. There was a wide dispersion of the quality assessment, particularly in the sound fragment assessment presented to the cavity ear.

## Discussion

We are the first to study habituation on the altered ear canal acoustics in patients with single sided cavities. Mean assessment of the naturalness of the sound fragment in the normal ear was in favour of the normal sound fragment. This is in line with our previous findings of quality assessment of the cavity acoustics presented to a normal ear [10]. Mean assessment of the naturalness of the sound fragment of the cavity ear presented to the cavity canal was close to baseline. This implies that our participants have comparable assessment of the naturalness of the cavity ear acoustics as to the normal ear canal acoustics, when presented to their cavity canal. This suggests that our participants habituate to their altered cavity ear canal acoustics. Our participants have had a cavity ear for many years (7–33 years). Possibly, over time our participants habituate to the acoustic properties of their cavity ear canal. In other fields of altered processing of sounds, habituation has been described before. Possible underlying neurophysiologic processes could be extrapolated from tinnitus studies. Perhaps, similar to the neurophysiologic model for habituation in tinnitus, some patients with a mastoid cavity evolve a more engaged attention system in the frontal cortex that suppresses the emotional response from the amygdala and other nodes of the limbic system [11].

Our participants asses sharpness of the sound fragment of the normal ear canal as clearly sharper if presented to both the normal and the cavity ear canal. We have previously demonstrated that acoustics of cavity canals amplify low to mid frequencies and reduce the soundwaves of high frequencies [6, 7, 10]. We had one outlier assessing sharpness of the cavity acoustics as more clear than the acoustics of the normal ear canal. A possible explanation could be, that for this individual participant the enhancement of the low- to mid-frequencies in the cavity canal improved the assessment of sharpness of the sound fragment. In our previous study, normal hearing subjects stated that the filtered sound fragment of cavity ear canals sound as if the sound fragment was ‘covered with a blanket’ [10]. Patients with a (dry) cavity ear canal benefit from hearing aids as much as non-operated ear canals [15]. Based on our findings, specific attention is needed for the altered acoustic properties of cavity ears in the hearing aid fitting process. Settings with a reduction of low- to mid-frequencies and enhancement of the high frequencies can restore the acoustic properties in the cavity canal. This should enhance sharpness and could help patients with cavity ear canals with improved discrimination and speech perception. Further research is needed to confirm this in patients with cavity ear canals. Surgical reconstruction of the ear canal can restore ear canal acoustics in cavity ears as well [6, 7]. Patients could experience improved sharpness of sound after canal wall reconstruction. Further research is needed to confirm this theory.

Overall assessment of the ear canal acoustics was in favour of the filtered sound fragment of the normal ear canal. This suggests that, although our participants seem to habituate to the altered ear canal acoustics of their cavity ear, the acoustics of the normal ear canal were generally deemed to be of superior sound characteristics. We think the improvement of sharpness of sound, due to the reduction of sound wave pressure of the low- to mid-frequencies and enhancement of the high frequencies, is accountable for the overall favour of the acoustics of the normal ear canal.

Due to our small sample size, we were unable to demonstrate statistically significant differences between the cavity ear canal assessment and the normal ear canal assessment of sound. Many of our candidate participants unfortunately failed to meet the inclusion criteria based on their PTA. Enhanced hearing thresholds would influence the assessment of acoustics and thereby bias our findings. Other candidate participants, referred to our tertiary center for revision cavity surgery with reconstruction of the posterior canal wall, failed inclusion because of the chronic infection of their cavity. Future research is needed to evaluate the patients’ perception on the adapted acoustics with hearing aids or after revision radical cavity surgery with canal wall reconstruction.

## Conclusions

Patients with cavity ear canals seem to habituate to their altered ear canal acoustics. Transforming the ear canal acoustics of the cavity ear to normal ear canal acoustics seem to improve the sharpness of the perceived sound fragment. Overall assessment of quality of sound is in favour of the normal ear canal acoustics.
